# Characterization of prostate cancer detected at repeat biopsy

**DOI:** 10.1186/1471-2490-8-14

**Published:** 2008-11-10

**Authors:** Takeshi Yuasa, Norihiko Tsuchiya, Teruaki Kumazawa, Takamitsu Inoue, Shintaro Narita, Mitsuru Saito, Yohei Horikawa, Shigeru Satoh, Tomonori Habuchi

**Affiliations:** 1 Department of Urology, Akita University School of Medicine, 1-1-1 Hondo, Akita 010-8543, Japan

## Abstract

**Background:**

The aim of this study was to investigate the characteristics of prostate cancer patients who were diagnosed at repeat biopsy and compare them to non-cancerous patients or patients who were diagnosed at initial biopsy.

**Methods:**

We carried out a retrospective analysis of clinical and pathological data from 576 patients, which included data on the period of time from radical prostatectomy to biochemical failure.

**Results:**

Cancer was diagnosed in 191 (33%) of 576 patients at initial biopsy and in 23 (18%) of 127 patients who underwent a repeat biopsy. Cut-off values of 0.80 and 0.30 for prostate specific antigen velocity (PSAV) and prostate specific antigen density (PSAD), respectively, were determined using ROC curve analysis. Based on these values, PSAV and PSAD were able to predict 94% (46 of 49) of negative repeat biopsies, indicating that these patients had undergone unnecessary repeat biopsies. Although the patients who were diagnosed at repeat biopsy had a higher rate of organ-confined tumor than those who were diagnosed at initial biopsy (73% and 44%, respectively; *P *= 0.041), there were no differences in the recurrence rate or the duration of biochemical failure-free survival between the two groups.

**Conclusion:**

PSAV and PSAD may be useful indicators of the results of repeat biopsies. Although prostate cancer that was diagnosed at repeat biopsy was associated with a more favorable pathological profile, it was not associated with a better outcome after radical prostatectomy.

## Background

Due to the wide-spread use of prostate specific antigen (PSA) screening, there has been an increasingly large number of men with elevated PSA and negative prostate biopsy [1]. A high percentage of these men undergo unnecessary repeat biopsy. In general, a repeat biopsy is indicated by increasing PSA levels, increased PSAD and PSAV, low free PSA ratio, and previous pathological findings, such as high grade intraepithelial neoplasia. However, there is currently no definitive or reliable predictor of repeat biopsy outcome. It is also largely unknown whether there are differences in the clinical and biological characteristics of prostate cancer that is detected at repeat biopsy as compared to initial biopsy [1].

Miyake et al. have reported that there was no significant difference in the pathological stage or volume of cancer diagnosed at initial biopsy as compared to repeat biopsy [2]. In contrast, Lopes-Corona et al. suggested that prostate cancer that is diagnosed at repeat biopsy is associated with favorable pathological findings at the time of radical prostatectomy. However, they also reported that there is a similar recurrence rate for cancers that are detected at repeat biopsy and initial biopsy [3]. These results suggest that the detection of prostate cancer at a repeat biopsy does not predict a favorable outcome. Rather, several studies have indicated that the number or percentage of positive biopsy cores may be an important predictor of a favorable outcome [4-7].

In the current study, we have assessed several conventional clinical variables associated with positive and negative repeat biopsies in order to identify a potential predictor of repeat biopsy outcome. We also compared the clinical and pathological characteristics and outcome associated with prostate cancer that was detected at initial and repeat biopsies.

## Methods

### Patient characteristics

Five hundred seventy-six (576) patients that were suspected of having prostate cancer underwent transrectal prostate needle biopsies from 1998 to 2006 at Akita University Medical Center. Of the patients who had a negative initial biopsy, 127 underwent a repeat biopsy. Six- or ten-transrectal biopsy cores were taken from the peripheral zone of the prostate using an 18-gauge needle biopsy gun under transrectal ultrasound guidance during the time periods of 1998–2003 or 2004–2006, respectively. For all patients with prostate cancer, clinical and pathological classifications were determined according to the World Health Organization criteria, the Gleason's histological grading, and the Tumor-Node-Metastatic system [8,9].

### Biochemical Failure

Biochemical failure was defined as a PSA level of greater than 0.2 ng/mL [10]. Patients were routinely seen for follow-up 1 month after prostatectomy, and every 6 to 12 months thereafter.

### Statistical analysis

The clinical values represent the means ± standard deviation (SD), and differences between groups were analyzed using the unpaired Student's *t*-test, Kruskal Wallis test, or the Mann-Whitney U test if the group variances were equal or non-normally distributed. Differences in clinical and pathological factors between groups were analyzed by the chi-square test. Receiver operating characteristic (ROC) curves were constructed by plotting sensitivity versus the false-positive rate. Biochemical failure-free survival time was calculated from the date of radical prostatectomy to the date of biochemical recurrence. Biochemical failure-free survival was estimated using the Kaplan-Meier method and differences in survival were analyzed using the logrank test. All data was entered into an Access database and analyzed by Excel 98 and SPSS (version 10.0J, SPSS Inc.) software programs. A probability (*P*) of < 0.05 was considered statistical significant.

## Results

### Clinical and pathological characteristics of patients with positive and negative repeat biopsies

The number of cores taken at repeat biopsy was greater than initial biopsy (7.31 ± 2.04 and 8.57 ± 2.11, respectively; *P *= 0.0034). Cancer was diagnosed in 191 of 576 patients (33%) at initial biopsy, and 23 of 127 patients (18%) who underwent repeat biopsy. The positive rate of diagnosis at initial biopsy was significantly higher than at repeat biopsy in our analysis (*P *= 0.042). There were no significant differences in age (72.5 ± 7.2 and 69.5 ± 7.2, *P *= 0.085), serum PSA level (12.6 ± 8.6 and 10.5 ± 7.5, *P *= 0.50), prostate volume (30.2 ± 23.6 and 39.7 ± 21.0, *P *= 0.15), PSAD (0.51 ± 0.43 and 0.34 ± 0.35, *P *= 0.13), PSAV (2.6 ± 6.2 and 0.83 ± 6.2, *P *= 0.16), or number of cores taken at repeat biopsy (8.6 ± 2.1 and 8.3 ± 2.3, *P *= 0.43) between patients who had positive and negative repeat biopsies, respectively. In addition, although we performed a multivariate logistic regression analysis that controls for this variable, we could not find a significant difference in these values between them.

### Analysis of PSA-related variables in patients with positive and negative results at repeat biopsy

**Figure 1:**
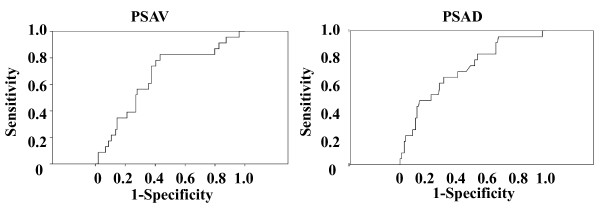
**ROC curves for PSAV and PSAD**. Receiver operating characteristic (ROC) curves were constructed by plotting sensitivity versus the false-positive rate using SPSS software.

We next examined the predictive value of PSAV and PSAD for repeat biopsy outcome. Using ROC curve analyses, we determined a cut-off value of 0.80 ng/ml/year and 0.30 ng/ml^2 ^for PSAV and PSAD, respectively (Figure 1). Based on this value, the sensitivity and specificity of PSAV for predicting a positive repeat biopsy was 78% and 63%, respectively (*P *= 0.0004, Table 1). Based on a cut-off value of 0.30 ng/ml^2^, the sensitivity and specificity of PSAD was 61% and 67%, respectively (*P *= 0.12, Table 1). The specificity and positive predictive value (PPV) of the combination of > 0.80 ng/ml/year PSAV and > 0.30 ng/ml^2 ^PSAD were 86% and 44%, respectively (Table 1). Using these cut-off values, 44% (12 of 27) of patients would have been predicted to have a positive repeat biopsy. For patients with either > 0.80 ng/ml year PSAV or > 0.30 ng/ml^2 ^PSAD, the sensitivity and negative predictive value (NPV) were 87% and 94%, respectively (Table 1), and 44% (46 of 127) of patients would have avoided a repeat biopsy. However, 13% (3 of 23) of patients with prostate cancer would have been missed.

**Table 1:** Prediction of repeat biopsy results by PSAD and PSAV

	Cancer (n = 23)	Non-Cancer (n = 104)	*P*	sensitivity	specificity	PPV	NPV
PSAV (ng/ml year)			0.0004	78%	63%	32%	93%
≥ 0.80	18 (78%)	39 (37%)					
<0.80	5 (22%)	65 (63%)					
PSAD (ng/mL^2^)			0.12	61%	67%	29%	89%
≥ 0.30	14 (61%)	34 (33%)					
<0.30	9 (39%)	70 (67%)					

PSAV ≥ 0.80 and PSAD ≥ 0.30	12 (52%)	15 (14%)	6.2 × 10^-5^	52%	86%	44%	89%
PSAV ≥ 0.80 and PSAD < 0.30	6 (26%)	24 (23%)					
PSAV < 0.80 and PSAD ≥ 0.30	2 (9%)	19 (18%)					
PSAV < 0.80 and PSAD < 0.30	3 (13%)	46 (44%)	0.0054	87%	44%	26%	94%

Abbreviations: PSA, prostate specific antigen; PSAD, PSA density; PSAV, PSA velocity; PPV, positive predictive value; NPV, negative predictive value.

### Clinical and pathological characteristics of patients diagnosed with prostate cancer at initial and repeat biopsy

**Figure 2:**
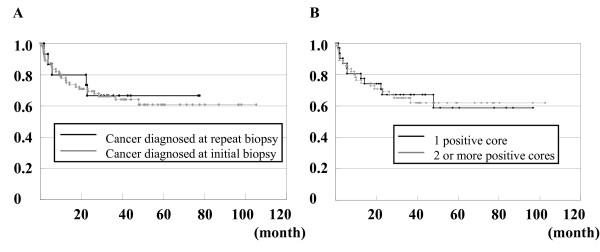
**Biochemical failure-free survival curves after radical prostatectomy**. Biochemical failure-free survival curves for patients whose cancer was detected at an initial biopsy and a repeat biopsy (A). Biochemical failure-free survival curves for patients with one positive core and two or more positive cores at biopsy (B).

We next compared the clinical and pathological characteristics of patients who were diagnosed with prostate cancer an initial or repeat biopsy. In order to avoid the selection bias, twenty-nine of the patients with distant metastasis, who were diagnosed at initial biopsy, were excluded in this analysis. As shown in Table 2A, patients who were diagnosed with prostate cancer at a repeat biopsy had significantly higher rates of non-palpable and organ-confined disease than patients who were diagnosed at an initial biopsy (Table 2A). Ninety-three and 9 patients who were diagnosed at initial biopsy and seventeen and 2 patients who were diagnosed at repeat biopsy underwent radical prostatectomy and local external beam radiation therapy, respectively. Among these patients, the clinical and pathological variables of 72 patients diagnosed at initial biopsy and 15 patients diagnosed at repeat biopsy, who underwent radical prostatectomy without neoadjuvant or adjuvant therapy, were compared (Table 2B). We then compared the post-radical prostatectomy clinical and pathological variables of patients who were diagnosed at initial and repeat biopsy. The mean period of follow-up for patients who were diagnosed at initial and repeat biopsy was 35 ± 26 (range 1 to 105) and 33 ± 22 (range 2 to 78) months, respectively. This difference was not statistically significant (*P *= 0.68). There was significantly more organ-confined disease detected at repeat biopsy than at initial biopsy (*P *= 0.041, Table 2B). However, the rate of biochemical failure and the duration of biochemical failure-free survival were not different between the two groups (Figure 2A). These results suggested that prostate cancer patients who are diagnosed at a repeat biopsy do not have better outcomes than those diagnosed at an initial biopsy, although the patients diagnosed at repeat biopsy had more favorable pathological findings in the tissues that were removed by radical prostatectomy. However, it is worth noting that the number of patients analyzed in this study was not sufficient to make this a conclusive finding.

**Table 2:** Characteristics of prostate cancer

**A **Clinical characteristics of prostate cancer diagnosed at repeat and initial biopsy.			
	Cancer at repeat biopsy (n = 23)	Cancer at initial biopsy (n = 162)	*P*

Patient age*	72.0 ± 5.7	71.5 ± 6.8	0.670
PSA (ng/ml)*	12.6 ± 8.6	27.0 ± 38.8	0.205
Gleason score*	6.3 ± 2.0	7.11 ± 1.59	0.150
No. of positive cores*	2.6 ± 2.2	3.33 ± 2.25	0.204

cT			0.005
T1a-c	18 (78%)	79 (49%)	
T2a-c	5 (22%)	61 (37%)	
T3a,b	0 (0%)	16 (10%)	
T4	0 (0%)	6 (4%)	

cN			0.059
N0	23 (100%)	142 (79%)	
N1, N2	0 (0%)	20 (21%)	

**B **Pathological characteristics of prostate cancer diagnosed at repeat biopsy and initial biopsy at the time of radical prostatectomy.			

	Cancer at repeat biopsy (n = 15)	Cancer at initial biopsy (n = 72)	*P*

Patient age*	72.3 ± 6.4	68.5 ± 5.5	0.042
PSA level*	14.3 ± 9.5	16.4 ± 24.9	0.59
Gleason score*	7.2 ± 1.4	7.1 ± 1.7	0.81

Pathological factor			
≤ pT2b	11 (73%)	32 (44%)	0.041
≥ pT3a	4 (27%)	40 (56%)	

pN0	14 (93%)	67 (93%)	0.97
pN1	1 (7%)	5 (7%)	

cap (+)	4 (27%)	32 (44%)	0.20
pn (+)	6 (40%)	37 (51%)	0.42
sv (+)	1 (7%)	12 (17%)	0.32

Biochemical failure	4 (27%)	26 (36%)	0.48

*Data indicates the means ± SD.

^#^Biochemical failure represents the number of the patients, who were diagnosed as biochemical failure.

Abbreviations: PSA, prostate specific antigen; cap(+), positive for capsular invasion; pn(+), positive for perineural invasion; sv(+), positive for seminal vesicle invasion.

### Clinical and pathological characteristics of patients diagnosed with prostate cancer with one positive core, or two or more positive cores at biopsy

To determine whether the number of positive cores obtained by biopsy was clinically significant, we compared the clinical and pathological characteristics of patients following radical prostatectomy who had one positive core or two or more positive cores at biopsy. The mean period of follow-up for patients with one positive core and those with two or more positive cores was 31 ± 22 (range 1 to 98) and 37 ± 22 (range 1 to 105) months, respectively. This difference was not statistically significant (*P *= 0.45). Compared to patients who had two or more positive cores, patients who had only one positive core had significantly lower serum PSA levels, less pathological organ-confined disease, and a lower rate of positive perineural infiltration and lymph duct invasion (Table 3). However, the rate of biochemical failure and duration of biochemical failure-free survival were not different between the two groups (Figure 2B). These results indicated that the number of positive cores at biopsy is not a good predictor of disease progression following radical prostatectomy.

**Table 3:** Clinical and pathological characteristics of patients who underwent radical prostatectomy with one positive core, and two or more positive cores at biopsy

	One positive core (n = 31)	Two or more positive cores (n = 56)	*P*
Age*	69.1 ± 5.6	68.8 ± 5.2	0.67
PSA*	12.5 ± 9.4	15.2 ± 14.0	0.021
Gleason score*	6.8 ± 1.6	7.3 ± 1.6	0.20
No. of positive cores*	1	3.7 ± 1.9	1.3 × 10^-16^

Pathological factor			0.038
≤ pT2b	21 (57%)	25 (44%)	
≥ pT3a	10 (43%)	31 (56%)	

pN0	30 (97%)	51 (91%)	0.31
pN1	1 (3%)	5 (9%)	

cap (+)	10 (32%)	28 (44%)	0.11
pn (+)	8 (26%)	35 (51%)	0.0001
sv (+)	2 (7%)	12 (17%)	0.069
PSA failure	10 (32%)	20 (36%)	0.75

*Data represents the means ± SD.

Abbreviations: as described for Table 2B.

## Discussion

Several studies have been performed, mainly in Western countries, to identify the risk factors for cancer in men undergoing repeat prostate biopsy [11-13]. Borboroglu et al. demonstrated that the only statistically significant predictor of a positive repeat biopsy was PSAV (*P *< 0.001). Prostate cancer was detected in 64% of men with a PSAV of 1 ng/ml or higher in extensive transrectal ultrasound guided prostate biopsies (average of 22.5 cores) [11]. Djavan et al. have suggested that percent-free PSA was the most accurate predictor of prostate cancer in settings where a repeat biopsy was performed [12]. The authors carried out a large retrospective study, and concluded that repeat biopsies should be performed in patients with a percent free PSA of less than 30% or a transition zone PSAD of 0.26 ng/ml^2 ^or greater [12]. Ouyang et al. reported that the presence of atypia at initial biopsy is a strong predictor of malignancy in subsequent biopsies [13]. On the other hand, in a Japanese study, Park et al. reported that total PSA, PSAD, PSAV, digital rectal examination, and TRUS findings were independent predictors of a positive repeat biopsy [14]. Despite these data, there is currently no standard set of criteria governing the performance of repeat biopsies following a negative initial biopsy in Japan or in Western countries. In the current study, we have carried out a retrospective analysis of clinical and pathological data on positive and negative repeat biopsies in order to identify predictors of repeat biopsy outcome. Based on an ROC curve analysis, we determined cut-off values for PSAV and PSAD of 0.80 ng/ml/year and 0.30 ng/ml^2^, respectively. When at least one of these criteria was satisfied, we predicted 87% (20 of 23) of positive repeat biopsies. We also determined that 94% (46 of 49) of patients who had PSAV and PSAD values that were below the cut-off value underwent unnecessary repeat biopsies. Furthermore, the use of PSAV and PSAD criteria would have spared 44% (46 of 127) of patients from undergoing a repeat biopsy (Table 1). These results warrant additional studies to identify other variables that can be used in conjunction with PSAV and PSAD to predict the results of a repeat biopsy, and decrease the number of needless repeat biopsies performed.

We also carried out a retrospective analysis of the clinical and pathological characteristics and outcomes of patients who were diagnosed at initial and repeat biopsies. Miyake et al. demonstrated that there were no significant differences in the final pathological features of prostate cancers that were detected at initial and repeat biopsies [2]. Although the authors did not analyze the outcome of patients who were diagnosed at repeat biopsy, they speculated that the biological behavior of the tumors that are detected at initial and repeat biopsies may be similar. In the current study, we found that patients who were diagnosed at a repeat biopsy had a significantly lower pathological T-stage than those who were diagnosed at an initial biopsy. However, the outcome after radical prostatectomy was similar between the two groups (Figure 2A). We also found that the number of positive cores at biopsy was not predictive of outcome after radical prostatectomy (Figure 2B).

There have been several recent studies evaluating the rate or duration of biochemical recurrence-free survival after radical prostatectomy in patients who are diagnosed at repeat biopsy. The largest retrospective study was carried out by Lopez-Corona et al. [3]. The authors found that cancer was diagnosed in 1,042 patients at an initial biopsy and 315 at a repeat biopsy. Patients who were diagnosed at repeat biopsies and underwent radical prostatectomy had a higher rate of clinical T1c stage cancer and organ-confined disease than patients who were diagnosed at an initial biopsy (*P *< 0.0001) [3]. However, despite the appearance of more favorable pathological features in tumors that were detected at a repeat biopsy, there was no difference in biochemical recurrence rate [3]. Steiner et al. demonstrated that when prostate cancer was diagnosed at a repeat biopsy, a negative result at the initial needle biopsy was predictive of a lower pathologic stage and grade, as well as smaller tumor volume [15]. However, patients who were diagnosed at a repeat biopsy did not have more favorable outcomes after radical prostatectomy. Our results agree with these previous studies, and indicate that patients who are diagnosed at a repeat biopsy include those with clinically insignificant and organ-confined cancer, and those with treatment delay. To our knowledge, this is the first study to demonstrate that biochemical recurrence-free survival after radical prostatectomy is similar in an Asian population of prostate cancer patients who were diagnosed at initial or repeat biopsies, similar to Caucasian populations.

The average number of cores per biopsy increased over the period of time examined in the current study, from 6 cores (between 1998 and 2003) to 10 cores (between 2004 and 2006). In addition, in the later period, there were more patients diagnosed with prostate cancer at a repeat biopsy (90/191 positive initial biopsy, 21/23 positive repeat biopsies). These variables represent a potential for bias in the current study, in terms of both the pathological features of the tumor and prognosis. However, our results warrant additional comprehensive analyses of patients that are diagnosed with prostate cancer at repeat biopsies.

## Conclusion

We have shown that a PSAV of ≥ 0.80 ng/ml/year and a PSAD of ≥ 0.30 ng/ml^2^may be useful criteria for predicting the result of a repeat biopsy, although in the current analysis, a considerable number (approximately 13%) of positive repeat biopsies were missed. Additional studies are needed to identify other variables that can be used in conjunction with PSAV and PSAD to predict the results of repeat biopsies. In addition, although we found that when cancer was detected at a repeat biopsy it was associated with favorable pathological findings, it was not associated with a better outcome following radical prostatectomy.

## Competing interests

The authors declare that they have no competing interests.

## Authors' contributions

All authors participated in study design; TY, NT, and TK carried out data collection, data interpretation, and drafted the manuscript. TY, TI, SN, and SS performed statistical analysis and literature search. TH performed a critical review of the manuscript and has given final approval of the version to be published. All authors read and approved the final manuscript.

## Pre-publication history

The pre-publication history for this paper can be accessed here:


